# Vitamin D status in women with dichorionic twin pregnancies and their neonates: a pilot study in China

**DOI:** 10.1186/s12884-021-03707-7

**Published:** 2021-04-08

**Authors:** Xin Li, Jiaxiao Yu, Li Wen, Qingshu Li, Jianying Yan, Jing Tian, Chao Tong, Qi Tong, Hongbo Qi, Richard Saffery, Mark D. Kilby, Philip N. Baker

**Affiliations:** 1 Department of Obstetrics, The First Affiliated Hospital of Chongqing Medical University, 1 Youyi Road, Yuzhong District, Chongqing, 400016 China; 2 State Key Laboratory of Maternal and Fetal Medicine of Chongqing Municipality, Chongqing Medical University, Chongqing, 400016 China; 3 International Collaborative Laboratory of Reproduction and Development of Chinese Ministry of Education, Chongqing Medical University, Chongqing, 400016 China; 4 Department of Pathology, The First Affiliated Hospital of Chongqing Medical University, Chongqing, 400016 China; 5 Fujian Provincial Maternity and Children’s Hospital, affiliated hospital of Fujian Medical University, Fuzhou, 350001 Fujian China; 6 Department of Obstetrics and Gynecology, University-Town Hospital of Chongqing Medical University, Chongqing, 401331 China; 7 Chongqing Population and Family Planning Science and Technology Research Institute, 18 Honghuang Rd, Jiangbei District, Chongqing, 400020 China; 8 Cancer, Disease and Developmental Epigenetics, Murdoch Children’s Research Institute, Parkville, Victoria 3052 Australia; 9 Centre for Women’s and Newborn Health, Institute of Metabolism and Systems Research, University of Birmingham, Birmingham, B15 2TT UK; 10 Fetal Medicine Centre, Birmingham Women’s & Children’s Foundation Trust, Edgbaston, Birmingham, B15 2TG UK; 11 College of Life Sciences, University of Leicester, Leicester, LE1 7RH UK

**Keywords:** Vitamin D, Twin pregnancies, Neonates, Prospective study, Nutrition

## Abstract

**Background:**

Vitamin D deficiency is a global public health issue in women and children and is associated with adverse impacts on child growth, such as rickets. However, prior studies have mainly focused on measuring vitamin D levels in singleton pregnant women and their offspring, and very limited studies have revealed the prevalence of vitamin D deficiency in twin pregnant women and their offspring. The aim of this study was to investigate vitamin D levels in twin-pregnant women and their neonates. We also explored the correlation of maternal vitamin D levels with neonatal outcomes and infant growth.

**Methods:**

A prospective subcohort investigation was carried out among 72 dichorionic, diamniotic twin-pregnant mothers and their twin offspring from the Longitudinal Twin Study. Peripheral blood was collected from the mothers in the third trimester, and cord blood was collected from neonates at birth to identify 25[OH]D levels. Data on the characteristics of the mothers and neonates were collected. Infant growth data and food sensitivities were also collected.

**Results:**

The average maternal 25[OH]D level was 31.78 ng/mL, with 19.4% being deficient and 20.8% insufficient, while the average neonatal 25[OH]D level was 15.37 ng/mL, with 99.3% being deficiency or insufficient. A positive correlation was found between maternal and neonatal 25[OH]D levels (beta-value: 0.43, 95% CI: 0.37, 0.49). Interestingly, the higher the maternal 25[OH]D level was, the smaller the cotwin birthweight discordance (beta-value: -2.67, 95% CI: − 5.11, − 0.23). In addition, the infants of mothers with vitamin D deficiency were more likely to be allergic to foods at 6 months than those of mothers with vitamin D sufficiency.

**Conclusions:**

Twin neonates were at high risk of vitamin D deficiency, although their mothers’ vitamin D deficiency partially improved. Higher maternal vitamin D levels were associated with smaller discordance of cotwin birthweight.

**Trial registration:**

Chinese Clinical Trial Registry ChiCTR-OOC-16008203, 1st April 2016.

**Supplementary Information:**

The online version contains supplementary material available at 10.1186/s12884-021-03707-7.

## Background

Vitamin D is a potent steroid hormone that is not only essential for building and maintaining bones but also plays an important role in the immune, endocrine and cardiovascular systems [1–4]. Although both animal-derived and plant-derived food sources can provide some vitamin D, the main source of vitamin D in the body depends on exposure to sunlight [5, 6]. However, the amount of time that people spend in the sun in modern society is not enough to meet their vitamin D needs [7, 8], especially since excessive ultraviolet radiation causes skin problems. In addition, ethnicity, latitude and body mass index also influence vitamin D status [9–11].

Vitamin D deficiency is a global public health problem, especially among pregnant women and newborns [12, 13]. A deficiency of vitamin D in pregnant women increases the risk of gestational diabetes, gestational hypertension disorder and insufficient gestational weight gain and may affect fetal growth and bone ossification [14, 15]. Patients with vitamin D deficiency have been shown to have a higher risk of calcemia and respiratory distress syndrome and a higher likelihood of developing food sensitivities, asthma, type I diabetes or autism later in life [16–20]. Previous studies have revealed that vitamin D status in the fetus and newborn is largely dependent on maternal vitamin D status; thus, the main risk factor for newborn vitamin D deficiency is maternal vitamin D deficiency [21–23]. However, the data on vitamin D status among twin-pregnant women and their offspring are very limited.

In recent decades, the prevalence of twin pregnancies has increased 1.8-fold according to the National Vital Statistics Report of the US due to the development of assisted reproductive technology and advanced maternal age [24–26]. In comparison with those with singleton pregnancy, women who are pregnant with twins are considered to undergo more complex physiological changes and obviously have a higher risk of adverse obstetric consequences [27, 28]. More attention should be paid to twin pregnancies with respect to nutrition and vitamin supplementation. Thus, it is essential to clarify whether vitamin D deficiency in mothers and newborns worsens among twin pregnancies.

Therefore, in the present study, we aimed to investigate the status of 25[OH]D in mothers and their newborns in a twin pregnancy and birth cohort from Southwest China. Given the previous findings reported for singleton pregnancy, we also aimed to determine the impact of maternal 25[OH]D deficiency on maternal and neonatal outcomes as well as child growth.

## Methods

### Study design and participants

The present study was embedded in the Longitudinal Twin Study (LoTiS), an ongoing twin pregnancy and birth cohort study conducted in Chongqing, which aims to determine the relative contributions of genes and the environment to early-onset intermediate phenotypes related to later adult onset disease. Chongqing is situated in southwestern China at a latitude of 29.35° N and characterized by a subtropical monsoon humid climate. This city has insufficient sunshine (1000–1400 h/year), especially in winter and spring. The LoTiS twin cohort study was established in January 2016 and aims to unravel the complex interplay between genes and the environment in specifying early life determinants of illness in infancy (Chinese Clinical Trial Registration Number: ChiCTR-OOC-16008203) and was approved by the Ethics Committee of the First Affiliated Hospital of Chongqing Medical University (No. 201530). Written informed consent was obtained from all participants. This subgroup study included dichorionic twin pregnant women confirmed prenatally by ultrasound with a prepregnancy BMI in the normal range (18.5 ~ 23.9) as well as daily multivitamin (vitamin D: 500 IU) supplementation from the first trimester to delivery. Due to the high incidence of adverse outcomes in monochorionic twin pregnancies, they were excluded from this study. Therefore, this study investigated vitamin D status in dichorionic diamniotic twin pregnancies. In addition, previous studies have shown that obese pregnant women may have abnormal vitamin D intake [29, 30], so we only included pregnant women with normal prepregnancy BMI. Mothers with chronic metabolic diseases and those using immunosuppressants were excluded. Twin pairs with a birth weight < 1500 g, significant malformations, or genetic disorders were also excluded. All twin offspring received daily usage of vitamin D supplements (400 IU/day) from birth and had a pediatric follow-up visit thereafter at a corrected age of 6 months.

### Data collection

Maternal sociodemographic data (age, height, weight, education, occupation, parity, mode of conception), lifestyle behaviors before pregnancy (smoking and alcohol use) and pre-existing conditions were collected by standardized questionnaire in the first follow-up during 11–16 gestational weeks. The standardized questionnaire was self-designed for the LoTiS cohort study, and detailed information is presented in Supplemental File 1. Information about vitamin D supplementation and other nutrients was collected using a structured questionnaire during the third trimester. Detailed information on this questionnaire is presented in Supplemental File 2. Pregnancy complications and maternal and neonatal outcomes, including gestational age, preterm premature rupture of membranes (PPROM, < 37 gestational weeks), neonatal sex, birthweight and small-for-gestational age (SGA, defined as a weight below the 10th percentile for GA and sex [31]), were collected from medical records.

### Measurement of serum 25[OH]D and classification criteria

Peripheral blood samples were collected from the mothers in the third trimester, and cord blood samples were immediately collected from newborns after placental delivery by using a coagulation-promoting blood collection tube. The specimen was transported to the Maternal and Fetal Medical Laboratory under refrigeration, where they were centrifuged to obtain serum. The serum 25[OH]D level was measured by high-performance liquid chromatography-electrospray tandem mass spectrometry (HPLC-MS-MS, Waters, USA), which is the gold standard method [32]. The concentrations of 25[OH]D_3_ and 25[OH]D_2_ were measured separately, and the total level of 25[OH]D was the sum of 25[OH]D_3_ and 25[OH]D_2._

Serum 25[OH]D is the best estimator to assess body vitamin D levels in the body [33]. For analysis, we divided mothers into 3 groups based on 25[OH]D levels: 25[OH]D levels < 20 ng/mL indicated deficiency, 25[OH]D levels in the range of 20–30 ng/mL indicated insufficiency, and 25[OH]D levels > 30 ng/mL were considered sufficiently high [34]. The same cutoffs were used for neonates according to the Chinese standard [35].

### Infant growth assessment and skin prick test

Infant growth was monitored for weight and length at the corrected age of 6 months. The corrected age was defined as the infant’s chronological age minus the difference between term birth (40 weeks) and chronological gestational weeks of delivery. Weight and length were simultaneously measured unclothed by a digital measuring bed (Beideneng, Shanghai, China) operated by trained nurses at the Department of Child Health Care of Chongqing Health Center for Women and Children. The indexes of weight, height and BMI were converted to Z-scores for sex and age according to WHO Child Growth Standards software (https://www.who.int/childgrowth/software/en/).

On the same day, a skin prick test (SPT) was performed to preliminarily diagnose food allergies. The SPT was performed on the volar surface of the forearm with a lancet through the use of a drop of an allergen extract, and test results were produced in 15 to 20 min. The routine test screened for food types including milk, egg white, egg yolk, peanut, fish, wheat, soybean, citrus, and apple.

### HPLC-MS-MS

The mass spectrometry conditions were an electrospray ionization source with multiple reaction ion scanning modes. Voltages: capillary (3 kV), cone (20 V), source offset (50 V), temperature (400 °C). GAS Flow: Desolvation (800 L/Hr), Cone (150 L/Hr), Nebulizer (7.0 The calibration curve showed good linearity over the range of 1–80 ng/mL for 25[OH]D_2_ and 4–200 ng/mL for 25[OH]D_3_, with <±15% RSD and RE. The intra-assay and inter-assay coefficients of variation were < 15% in all assays, indicating good repeatability.

### Statistical methods

All statistical analyses were performed with SPSS version 25.0 (IBM, Armonk, NY, USA). Categorical variables are presented as the count and percentage and were analyzed by the chi-squared or Fisher’s exact test. Continuous variables are presented as the means and standard deviation and were analyzed by Student’s t-test, LSD Student’s t test, one-way analysis of variance or the nonparametric test. Linear correlation analysis was used to explore the correlation between 25[OH]D levels in mothers and newborns and the correlation of 25[OH]D levels between cotwins. Multivariable linear regression analysis was used to detect the associations between neonatal 25[OH]D levels and related clinical characteristics. All tests were two-tailed, and *p* < 0.05 was considered statistically significant.

## Results

**Fig. 1:**
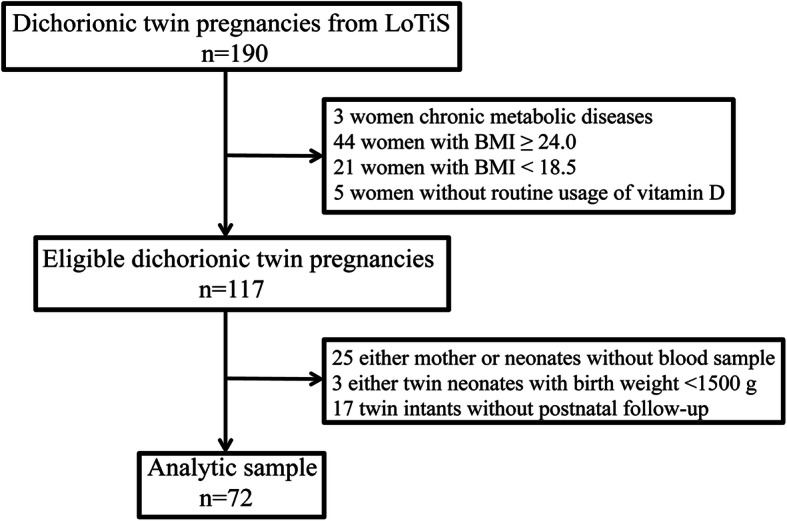
The selection process for this study

The selection process for this study population is presented in Fig. 1. A total of 190 dichorionic twin pregnancies from LoTiS were initially recruited into this subgroup study. After excluding mother-twin offspring pairs that did not meet the inclusion criteria and did not have blood samples, a total of 72 mother-twin offspring pairs were available for the final analysis. The descriptive data of the study participants are shown in Table 1. The average maternal age at recruitment was 30.46 ± 2.93 years, and the average gestational age was 36.77 ± 1.16 weeks. The mean birth weight of all neonates was 2626.53 ± 329.96 g. In addition, 62.5% of mothers conceived with the aid of assisted reproductive technology (ART), and 66.7% of mothers delivered in the summer or autumn. The average maternal 25[OH]D level was 31.78 ± 11.1 ng/mL, with 19.5% of mothers being deficient and 20.8% being insufficient. Unexpectedly, the average neonatal 25[OH]D level was 15.37 ± 4.86 ng/mL, with 78.5% of neonates being deficient and 20.8% being insufficient.

**Table 1:** Description of the maternal and neonatal characteristics

Variables	Total
Mothers	72
Age (year)	30.46 ± 2.93
Pre-pregnancy BMI (kg/m2)	21.15 ± 1.32
Mode of conception	
Natural conception	27 (37.5%)
Assisted reproductive technology	45 (62.5%)
Gestational weight gain (kg)	17.65 ± 5.89
Gestational age (week)	36.77 ± 1.16
Preterm birth	33 (45.8%)
Sampling season	
Summer/autumn	24 (33.3%)
Winter/spring	48 (66.7%)
25[OH]D level (ng/mL)	31.78 ± 11.1
Deficiency (< 20 ng/mL)	14 (19.5%)
Insufficiency (20-30 ng/mL)	15 (20.8%)
Sufficiency (> 30 ng/mL)	43 (59.7%)
Infants	144
Gender (male)	70 (48.6%)
Birthweight (g)	2626.53 ± 329.96
SGA	5 (3.5%)
25[OH]D level (ng/mL)	15.37 ± 4.86
Deficiency (< 20 ng/mL)	113 (78.5%)
Insufficiency (20-30 ng/mL)	30 (20.8%)
Sufficiency (> 30 ng/mL)	1 (0.7%)

In the maternal vitamin D deficiency group, over half of the mothers conceived with ART and were complicated with gestational diabetes, and 78.6% delivered in the winter or spring; this group had the highest incidence of SGA, with the lowest birthweight as well as the highest birthweight discordance (Table 2).

**Table 2:** Description of the maternal and neonatal characteristics by maternal vitamin D status

Variables	Deficiency	Insufficiency	Sufficiency	*p*-value
Mothers	*N* = 14	*N* = 15	*N* = 43	
Age (year)	29.86 ± 3.57	29.87 ± 3.54	30.86 ± 3.52	0.394^a^
Pre-pregnancy BMI (kg/m2)	20.76 ± 1.30	20.50 ± 1.26	21.50 ± 1.62	0.054^a^
Mode of conception				0.868^b^
Natural conception	6 (42.9%)	5 (33.3%)	16 (37.2%)	
ART	8 (57.1%)	10 (66.7%)	27 (62.8%)	
Pregnancy weight gain (kg)	17.50 ± 4.97	19.43 ± 4.44	17.07 ± 6.55	0.412^a^
Gestational age (week)	36.62 ± 0.97	36.98 ± 1.08	36.73 ± 1.24	0.473^a^
Preterm birth	7 (50.0%)	6 (40.0%)	20 (46.5%)	0.856^b^
Pregnancy-induced illness				
GDM	8 (57.1%)	6 (40.0%)	12 (27.9%)	0.133^b^
GHD	1 (7.1%)	1 (6.7%)	2 (4.7%)	0.510^c^
ICP	4 (28.6%)	0 (0%)	8 (18.6%)	0.919^c^
Sampling season				0.538^b^
Summer/autumn	3 (21.4%)	6 (40.0%)	15 (34.9%)	
Winter/spring	11 (78.6%)	9 (60.0%)	28 (65.1%)	
Infants	*N* = 28	*N* = 30	*N* = 86	
Gender (male)	12 (42.9%)	19 (63.3%)	39 (45.3%)	0.188^b^
Birthweight (g)	2453 (365)	2740(274)	2641 (347)	0.001^d^
Birthweight discordance (%)	8.83 (1.21)	6.93 (3.87)	5.72 (4.06)	0.048^d^
SGA	2 (7.1%)	1 (3.3%)	2 (2.3%)	0.481^c^
25[OH]D level (ng/mL)	7.93 (6.20)	12.89 (6.34)	18.75 (5.46)	< 0.001^d^

*BMI* body mass index, *ART* assisted reproductive technology, *GDM* gestational diabetes, *GHD* gestational hypertension disorder, *ICP* intrahepatic cholestasis of pregnancy, *SGA* small for gestational age

^a^Average and standard deviation. One-way Analysis of Variance

^b^Number (percentage). Chi-squared Test

^c^Number (percentage). Fisher Exact Test

^d^Median (interquartile range). Kruskal-Wallis Test

**Fig. 2:**
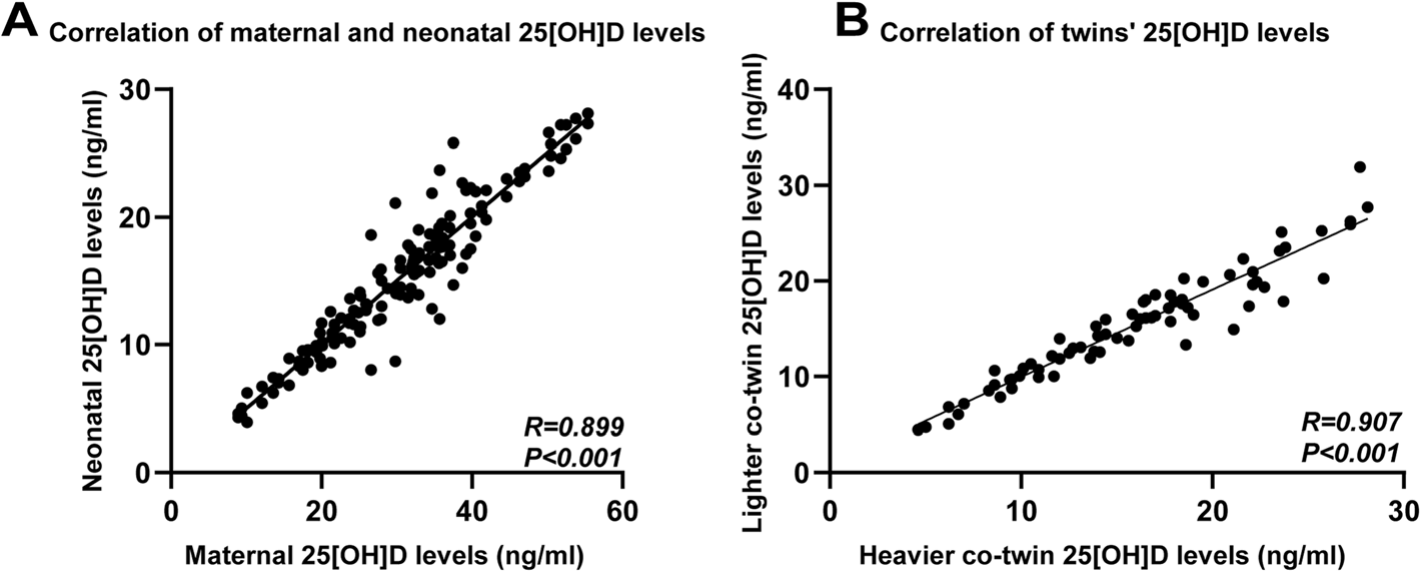
Correlation between maternal and neonatal 25[OH]D levels and correlation of cotwins’ 25[OH]D levels. **a** There was a directly proportional correlation between maternal 25[OH]D levels and neonatal 25[OH]D levels (*r* = 0.90, *p* < 0.001). **b** A significantly positive correlation was found between cotwins in terms of neonatal 25[OH]D levels (*r* = 0.91, *p* < 0.001)

A significant difference in neonatal 25[OH]D levels was found among the maternal vitamin D deficiency, insufficiency and sufficiency groups (Table 2). There was a directly proportional correlation between maternal 25[OH]D levels and neonatal 25[OH]D levels (*r* = 0.90, *p* < 0.001) (Fig. 2a). In addition, a significantly positive correlation was found between cotwins in terms of neonatal 25[OH]D level (*r* = 0.91, *p* < 0.001) (Fig. 2b). Multivariable linear regression showed that neonatal 25[OH]D levels were positively associated with maternal 25[OH]D levels (beta-value: 0.968, 95% CI: 0.459,0.531. *p* < 0.001) and birth season (beta-value: -0.102, 95% CI: − 1.013, 0.198. *p =* 0.004) and were not found to have a correlation with maternal age, BMI, gestational weight gain, gestational age, GDM, GHP, ICP or neonatal birthweight (Table 3).

**Table 3:** Association between pregnant covariates and neonatal 25[OH]D levels

Variables	Beta	95% CI	*P*-value
Maternal 25[OH]D level	0.968	(0.459,0.531)	< 0.001
Maternal age	−0.019	(−0.347,0.209)	0.622
Maternal pre-pregnancy BMI	−0.002	(−0.114,0.109)	0.965
Maternal gestational weight gain	0.015	(− 0.056,0.084)	0.684
Gestational age at delivery	−0.187	(−0.649,0.276)	0.423
Birth season	−0.102	(−1.013,-0.198)	0.004
Neonatal birth weight	−0.020	(−0.002,0.001)	0.672
GDM	−0.019	(−1.042,0.598)	0.591
GHD	0.029	(− 1.056,2.461)	0.427
ICP	−0.013	(− 0.522,0.399)	0.791

**Fig. 3:**
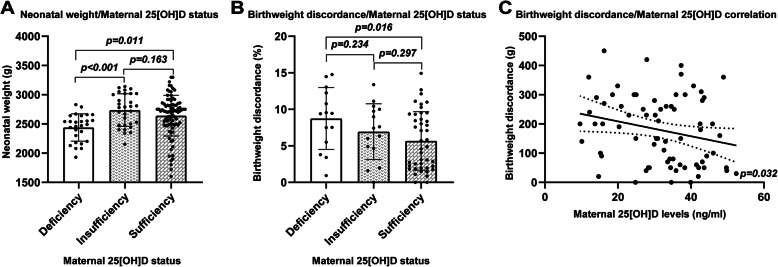
The impact of maternal 25[OH]D levels on neonatal birthweight. **a** The neonatal birthweight was significantly different between the maternal 25[OH]D status (deficiency group vs insufficiency group, *P* < 0.001; deficiency group vs sufficiency group, *P* = 0.011); **b** The neonatal birthweight discordance(%) was significantly different between the maternal 25[OH]D status (deficiency group vs sufficiency group, *P* = 0.016); **c** Maternal 25[OH]D levels were negatively correlated with the discordance in birthweight between cotwins, with the discordance in birthweight between cotwins decreased 2.67% when maternal 25[OH]D level increased by 1 ng/mL (95% CI: − 5.11, − 0.23. *p* = 0.032)

The neonatal birthweight and the discordance in birthweight between cotwins were significantly different between the maternal 25[OH]D deficiency and sufficiency groups (Fig. 3a-c). After adjusting for maternal age, BMI, GDM, GHP, ICP, gestational weight gain, gestational age and birth season, the results suggested that there was no correlation between maternal 25[OH]D levels and neonatal birthweight (Table 4). Surprisingly, maternal 25[OH]D levels were negatively correlated with the discordance in birthweight between cotwins, with the discordance in birthweight between cotwins decreasing by 2.67% when the maternal 25[OH]D level increased by 1 ng/mL (95% CI: − 5.11, − 0.23. *p* = 0.032) (Fig. 3c).

**Table 4:** Association between pregnant covariates and neonatal birthweight

Variables	Beta	95% CI	*P*-value
Maternal 25[OH]D level	0.392	(− 0.129,0.913)	0.112
Maternal age (years)	1.104	(−0.193,2.400)	0.139
Maternal pre-pregnancy BMI	−0.189	(−2.107,1.728)	0.095
Maternal gestational weight gain	0.007	(0.001,0.014)	0.050
Gestational age at delivery	1.768	(−2.079,5.615)	0.018
Birth season	−2.489	(−4.544,-0.434)	0.169
GDM	1.006	(−7.268,9.279)	0.365
GHD	5.398	(0.010,10.787)	0.810
ICP	−0.226	(−0.549,0.097)	0.845

At 6 months of corrected age, the z-scores of the weight-for-age, height-for-age, weight-for-height, and BMI-for-age indexes showed no differences among the maternal 25[OH]D deficiency, insufficiency and sufficiency groups for all infants taking vitamin D supplements each day. However, in the maternal vitamin D deficiency group, the incidence of allergies to foods was highest (Table 5).

**Table 5:** Description of the growth and food sensitivity by maternal 25[OH]D status

Variables	Deficiency (*N* = 28)	Insufficiency (*N* = 30)	Sufficiency (*N* = 86)	*p*-value
WHZ	0.39 ± 0.80	0.47 ± 0.73	0.35 ± 0.82	0.828^a^
WAZ	0.41 ± 0.88	0.59 ± 0.97	0.34 ± 0.85	0.527^a^
HAZ	0.29 ± 1.10	0.57 ± 1.21	0.42 ± 0.95	0.673^a^
BAZ	0.34 ± 0.66	0.38 ± 0.98	0.36 ± 0.79	0.981^a^
Allergic to one or more food	4 (14.3%)	2 (6.7%)	4 (4.7%)	0.200^b^

*WHZ* z-score for weight-for-height, *WAZ* z-score for weight-for-age, *HAZ* z-score for height-for-age, *BAZ* z-score for BMI-for-age

^a^Average and standard deviation. Student t test

^b^Number (percentage). chi-square test

## Discussion

In this prospective preliminary study, we reported that 19.5% of mothers had vitamin D deficiency, and their neonates had a remarkably high prevalence of vitamin D deficiency at birth, with a rate of 78.5%. We noticed very poor vitamin D stores, especially in twin neonates, even though all mothers took multivitamins (including vitamin D: 500 IU) daily during pregnancy.

Several studies conducted on singleton pregnancy among the Chinese population have investigated maternal 25[OH]D levels. Maternal vitamin D deficiency was reported in 79.2% of a multiethnic population without an investigation of prenatal vitamin D supplementation [36]. Approximately 10% of mothers took prenatal vitamin D, and the maternal vitamin D deficiency rate was also high at 74.9%. If the mothers took prenatal vitamin D daily during the last month before delivery or over three times per week, the maternal vitamin D deficiency rates improved to 36.6 and 31.6%, respectively [37]. The maternal vitamin D deficiency rate in this study was lower than that in the aforementioned studies. This can be explained by the fact that all subjects in this study took vitamin D supplements daily starting in the first trimester. Thus, we speculated that a high frequency of vitamin D supplementation during pregnancy is an effective way to reduce the risk of maternal vitamin D deficiency.

Previous studies have revealed that the rates of maternal and neonatal vitamin D deficiency are comparable in singleton pregnancy [36, 38]. A similar statement was also found in the northern Indian twin pregnancy population, with a maternal vitamin D deficiency rate of 90% and neonatal vitamin D deficiency rate of 89% [13]. However, the results of our study showed that the neonatal vitamin D deficiency rate was 4-fold higher than the maternal vitamin D deficiency rate, which was inconsistent with the aforementioned study. This is probably attributed to the distinct rates of maternal vitamin D deficiency in the two studies. The significant difference in neonatal vitamin D deficiency and maternal vitamin D deficiency incidence in our study was likely due to the maternal vitamin D supply to the two fetuses. In addition, we also found that the maternal vitamin D level was an independent factor that correlated with the neonatal vitamin D level, which was consistent with previous studies [39].

The clinical practice guidelines of the Endocrinology Society recommend that pregnant women take vitamin D supplements of at least 600 IU daily. However, twin neonates were at a very high risk of vitamin D deficiency when twin pregnant women took vitamin D supplements of 500 IU daily. Therefore, further investigations are needed to establish an appropriate dose that is effective for improving neonatal vitamin D deficiency and safe for maternal metabolism.

Consistent with previous studies [40, 41], we found that newborns born in winter may have lower 25[OH]D levels. Chongqing, also known as China’s fog capital, has few sunny days in winter. The long winter and lack of sunshine in Chongqing may be the cause of neonatal vitamin D deficiency.

There are conflicting reports about the association between maternal vitamin D levels and neonatal birthweight in singleton pregnancy. Hajianfar et al found a significant inverse association between maternal vitamin D level and the rate of low birthweight neonates [42]; others have reported no relationship [43, 44]. In our study, although the neonatal birthweight in the maternal vitamin D deficiency group was lower than that in the other group, no correlation was found between maternal vitamin D level and neonatal birthweight after adjusting for potential confounders. Interestingly, we found that the higher the maternal vitamin D level was, the smaller the discordance in birthweight between cotwins, thus presenting a negative association. However, further investigations are needed to detect the relative molecular mechanism.

Vitamin D deficiency in neonates has been shown to lead to a higher risk of food sensitivities later in life. In our study, because 99.3% of neonates were vitamin D deficient or insufficient, we compared the status of infants allergic to foods by maternal vitamin D levels. Although no significant difference was found among the maternal vitamin D deficiency, insufficiency and sufficiency groups in terms of allergies to foods, the allergy rate was highest in the maternal vitamin D deficiency group. It is worth exploring whether maternal vitamin D deficiency or neonatal vitamin D deficiency predominantly influences infant allergies to foods.

The strength of our study is the specialized study population. We used strict inclusion and exclusion criteria to screen the participants. Monochorionic twin pregnant women were not selected due to the higher risks of maternal and fetal complications, higher rate of preterm birth and lower neonatal birthweight. Prepregnancy BMI also has an obvious impact on perinatal outcomes, so we only recruited women with prepregnancy BMIs in the normal range. Additionally, the food sensitivities of infants at 6 months were followed, representing a relatively complete study design. Due to the low natural incidence of twin pregnancy [24], it is time-consuming to obtain participants. Therefore, ART twin pregnancies were also recruited to enlarge the sample size of this study. This also conferred us the opportunity to investigate whether ART has an impact on the 25[OH]D levels of twin pregnancies, and our data clearly revealed that ART does not disturb maternal and neonatal vitamin D levels in twin pregnancies.

This preliminary study contributes new knowledge about the status of maternal and neonatal vitamin D levels in twin pregnancies, but several limitations of this study should be taken into consideration. First, compared with that of similar studies in singleton pregnancy, the sample size in this study was relatively small. Second, pregnant women in our hospital were routinely advised to take multivitamin supplements daily based on the clinical guidelines, particularly twin pregnant women; thus, there were no women who did not take multivitamin supplements as controls. Third, it is more appropriate to measure vitamin D levels in women pregnant with twins in the first trimester, prescribe adequate dose supplements and monitor vitamin D levels during pregnancy. Then, the related variables of vitamin D metabolism were limited, such as dietary vitamin D intake and daily solarization not involved. Finally, singleton pregnant women taking the same multivitamin supplements and their neonates should be included as a control group. A large-scale study including singleton pregnancy and twin pregnancies conducted in multiple centers is essential to better understand the prevalence of maternal and neonatal vitamin D deficiency in China among twin pregnancy populations.

## Conclusions

In summary, this study suggested that despite twin-pregnant women taking prenatal vitamin D supplements and the mothers’ vitamin D deficiency partially improving, their twin neonates were at high risk of vitamin D deficiency. These findings indicated that obstetricians should pay special attention to the dose of vitamin supplements provided to the twin pregnancy population.

## Supplementary Information

**Additional file 1.**

**Additional file 2.**

## Data Availability

The LoTiS is being conducted mainly at the First Affiliated Hospital of Chongqing Medical University and Chongqing Women and Children’s Health Center, where the staff are responsible for the collection, management, and distribution of data. All data are stored electronically in an anonymous format and are currently only available to LoTiS researchers. The datasets used and/or analyzed during the current study are available from the corresponding author upon request (chaotongcqmu@163.com).

## Abbreviations

NC: Natural conception group
IVF: In vitro fertilization
BMI: Body mass index
CI: Confidence interval
GA: Gestational age
GDM: Gestational diabetes mellitus
ICP: Intrahepatic cholestasis of pregnancy
LoTiS: Longitudinal Twin Study
OR: Odds ratio
PE: Preeclampsia
SGA: Small for gestational age
ART: Assisted reproductive technology
GHD: Gestational hypertension disorder
WHZ: Z-score for weight-for-height
WAZ: Z-score for weight-for-age
HAZ: Z-score for height-for-age
BAZ: Z-score for BMI-for-age
