# Supplementary material for: Vitamin D status in women with dichorionic twin pregnancies and their neonates: a pilot study in China
Source: BMC Pregnancy Childbirth. 2021 Apr 8;21:279. doi: 10.1186/s12884-021-03707-7 (PMC8034067; doi:10.1186/s12884-021-03707-7)
Supplement: Supplementary file 1 — Additional file 1. [file 12884_2021_3707_MOESM1_ESM.docx]

**Supplemental File 1.** **Questionnaires for the LoTiS cohort study.**

# Questionnaires for the LoTiS cohort study

# Participant overview

Participant ID:

Center:

Midwife:

Registration date:

Recruitment date:

## Recruitment

### 1. Registeration

* Participant ID： * Participant’s name： *MW Contact Date： *Study ID：

### 2. Agreement

*I agree to take part in this study. Yes NO

1. I have been reading the informed consent, the researchers have explained the purpose, contents, risks and benefits of this research to me clearly. My questions so far have been answered. I understand the information printed on this form, and I volunteered for this study. Yes NO

2. I agree to donate my biological samples (including blood, urine, hair, nails, buccal smear) and my baby's biological samples and my husband's oral samples for this research. I know our biological samples such as blood, urine and hair could be sent to study abroad, and I know the donation is voluntary. I could withdraw from the study whenever I decide, which won’t affect the normal antenatal care in the First Affiliated Hospital of Chongqing Medical University. Yes NO

3. I agree the researchers to check on my medical records and personal information related to medicine. I know that my personal information will be kept secret. Yes NO

4. I agreed to the use of biological samples and personal information I donated in current and future scientific research (including commercial research and scientific research not related to this project). I know clearly that if this research could lead to new treatments and inventions of medical testing, I would not be able to gain commercial benefits from it. Yes NO

5. I have the opportunity to invite my family or friends to help me to ask questions about this study, I know the person I should contact with if there is a problem. Yes NO

### 3. Enrolment

Group： ①Monochorionic diamniotic cystic ②Dichorionic diamniotic cystic

③Monochorionic monochorionic cystic ④Multiple pregnancy

Inclusion Criteria：*Twin pregnancy or Multiple pregnancy

* Gestational ages no more than 16 weeks

***** Able to provide written, informed consent

Exclusion Criteria: *Death of One of Twin

* Malformation of one of twin

### 4. Pregnant woman basic information

*name：

*date of birth：

*native place：

*Telephone number：

*address：

* Patient ID：

*Prenatal examination file number：

*education：1- primary school 2- high school 3- University 4- university or above 5- illiterate 6- other:

*occupation：

*blood type：

*height： cm

*weight before pregancy： kg

*smoke（1-no 2- smoking before 3-still smoking, /d 4-History of passive smoking, years） *alcohol（1-no 2- drinking before 3-still drinking, g/week）

*husband’s name：

*age：

*height： cm

*blood type：

*education：1- primary school 2- high school 3- University 4- university or above 5- illiterate 6- other:

*occupation：

*telephone number：

### 5. Obstetric History

*Pregnancy（times）：_______（Including pregnancy, termination of pregnancy, abortion）

*Delivery（times）：_______

* Fetal malformations：①yes（add time）②no

*Premature delivery：①yes（add time） ②no

*Stillbirth：①yes（add time） ②no

*Tractus genitalis operation history（Cesarean section、obstetric forceps）：①yes（add time） ②no *Spontaneous abortion：①yes（add time）②no

*Artificial abortion：①yes（add time） ②no

*Odinopoeia：①yes（add time） ②no

*Acephalocystis racemosa：①yes（add time） ②no

* Gestational diabetes mellitus：①yes（add time） ②no

* Intrahepatic cholestasis of pregnancy：①yes（add time） ②no

* Pre eclampsia：①yes（add time） ②no

### 6. Current Pregnancy

* Last menstrual period：

* Gestational weeks：

*pregnancy mode：① Natural pregnancy ②IVF-ET ③ovulation stimulants

### 7. History of Present Illness

*Colporrhagia：①yes ②no

*Viral infection：①yes ②no

*Exposure to radiation：①yes ②no

*Hypermesis：①yes ②no

*Fever：①yes ②no

* Long term exposure to poison：①yes ②no

* Take medicine：①yes (add drug name and time) ②no

* Anemia：①yes(add type) ②no

* Take acyeterion before pregnancy 6 months：①yes（add drug name） ②no

other：

### 8. Past medical history

*Heart disease：①yes ②no

*High blood pressure：①yes ②no

*Nephritis：①yes ②no

*Hepatitis：①yes ②no

*Tuberculosis：①yes ②no

*Diabetes mellitus：①yes ②no

*Blood disease：①yes ②no

*Mental illness：①yes (add type) ②no

*Epilepsy：①yes ②no

*Thyroid dysfunction：①yes ②no

*Allergic history**：**①yes ②no

*Operation history：①yes（add operation name and time） ②no

Other：

### 9. Family History

*Twin history：①yes（add anyone）②no

* Gestational diabetes mellitus：①yes（add anyone）②no

* Intrahepatic cholestasis of pregnancy：①yes（add anyone）②no

* Pre eclampsia：①yes（add anyone）②no

*Neuropathy：①yes（add anyone）②no

*Dementia：①yes（add anyone）②no

*Malformation：①yes（add anyone）②no

*Genetic disease：①yes（add anyone）②no

*High blood pressure：①yes（mother or father） ②no

* Diabetes mellitus：①yes（mother or father） ②no

*Cardiovascular diseases：①yes（mother or father） ②no

* Immune diseases：①yes（mother or father） ②no

* Obstetric History：①yes（mother） ②no

*Tumor history：①yes（mother or father） ②no

Other：

### 10. Drug/vitamin supplement

|  | Which gestational weeks start taken | dose | frequency |
| --- | --- | --- | --- |
| Folic acid (mg) |  |  |  |
| Multi vitamin (mg) |  |  |  |
| Calcium (mg) |  |  |  |
| Iron (mg) |  |  |  |
| DHA |  |  |  |
| Aspirin (mg) |  |  |  |

Dose:0.01-100 mg frequency: tid, bid, qd, qod, qw
