# Supplementary material for: Vitamin D status in women with dichorionic twin pregnancies and their neonates: a pilot study in China
Source: BMC Pregnancy Childbirth. 2021 Apr 8;21:279. doi: 10.1186/s12884-021-03707-7 (PMC8034067; doi:10.1186/s12884-021-03707-7)
Supplement: Supplementary file 2 — Additional file 2. [file 12884_2021_3707_MOESM2_ESM.docx]

**Supplemental File 2. Questionnaires for** **vitamin D supplementation and other nutrients.**

# Questionnaires for the vitamin D supplementation and other nutrients

# Participant overview

Participant ID:

Center:

Midwife:

Registration date:

Recruitment date:

## Recruitment

### 1. Registeration

* Participant ID： * Participant’s name： *MW Contact Date： *Study ID：

### 2. Drug/vitamin supplement

| Variables | Answers |
| --- | --- |
| 1.Drug brand |  |
| 2.Drug name |  |
| 3.Which gestational weeks start taken |  |
| 4.Frequency |  |
| 5.Dose | (According to the drug brand, calculated by the investigator) |

Dose:0.01-100 mg frequency: tid, bid, qd, qod, qw
